# Understanding No-Show Patterns in Healthcare: A Retrospective Study from Northern Italy †

**DOI:** 10.3390/healthcare13151869

**Published:** 2025-07-30

**Authors:** Antonino Russotto, Paolo Ragusa, Dario Catozzi, Aldo De Angelis, Alessandro Durbano, Roberta Siliquini, Stefania Orecchia

**Affiliations:** 1 Department of Sciences of Public Health and Paediatrics, University of Turin, 10126 Turin, Italy; 2 S.C. Distretto Sud-Est, ASL Città di Torino, 10128 Turin, Italy

**Keywords:** patient appointments, utilization review, no-show patients, waiting lists, efficiency, organizational

## Abstract

**Objectives:** The aim of this study was to analyse no-show patterns in healthcare appointments, identify associated factors, and explore key determinants influencing non-attendance. **Study Design:** This was a retrospective observational study. **Methods:** We analysed 120,405 healthcare appointments from 2022–2023 in Turin, Northern Italy. Data included demographics, appointment characteristics, and attendance records. Logistic regression identified significant predictors of no-shows, adjusting for confounders. **Results:** A 5.1% (n = 6198) no-show percentage was observed. Younger patients (<18 years) and adults (18–65 years) had significantly higher odds of missing appointments than elderly patients (>65 years) (OR = 2.32, 95% CI: 2.17–2.47; OR = 2.46, 95% CI: 2.20–2.74; *p* < 0.001). First-time visits had a higher no-show risk compared to follow-up visits and diagnostics (OR = 1.11, 95% CI: 1.04–1.18; *p* < 0.001). Each additional day of waiting increased the likelihood of no-show by 1% (OR = 1.01, 95% CI: 1.01–1.01; *p* < 0.001). **Conclusions:** No-show percentages are influenced by demographic and service-related factors. Strategies targeting younger patients, longer waiting times, and non-urgent appointments could reduce no-show percentages.

## 1. Introduction

Every day, the public National Health Service (NHS) faces significant challenges in providing care, diagnosis, and specialized services to citizens [1,2]. However, the management of waiting lists (WLs) and appointment timings depends not only on assessing healthcare needs and developing supply plans but also on patient behaviour. The issue of no-shows for healthcare—instances where patients fail to attend scheduled appointments without notification of cancellation—significantly affect healthcare systems [3].

This behaviour has financial and operational implications for the NHS in Italy. For instance, outpatient services at the Local Health Authority (LHA) level involve user payments via a “ticket” fee when the patient is non-exempt. Failure to attend results in lost revenue, penalties, and administrative costs, such as staff time, IT management, and reminders. In Denmark, no-show fines are estimated to cost around DKK 40 (about EUR 5.4 in 2003 prices) [4].

The lack of communication around cancellations disrupts rescheduling efforts and prevents optimal use of healthcare resources and staff. This challenge compounds waiting-list problems by blocking access to potentially available slots [3,5,6].

No-shows are a global issue that is well documented in the literature, with various interventions and predictive models explored to address it [3,7,8].

A recent systematic review found an average no-show percentage of 19.3% in Europe; in Italy, a specialized healthcare centre reported a 14.6% no-show percentage [3,9].

Multiple countries have implemented interventions and predictive models to tackle no-shows: Predictive models can identify patients at high risk of no-show to enable targeted interventions such as personalized reminders and patient navigators, which have proven to be effective [10,11]. Moreover, patient education on the importance of attending appointments and engagement through personalized communication are identified as useful strategies to improve attendance rates [6]. Finally, other studies have focused on the possibility of implementing overbooking practices to address the no-show issue [12,13]. However, these practices should be based on a meticulous and accurate predictive model, carefully designed around the available healthcare data [9]. In Italy, significant measures to counter the phenomenon, including the “National Plan for the Management of WLs 2019–2021”, predate COVID-19 pandemic [14].

Regional Health Services (RHSs) have since introduced post-pandemic WL recovery plans, such as the Piedmont Region’s “Extraordinary Plan for the Recovery of WLs”, which allocated over EUR 36 million for LHA-level initiatives. These funds could have been used for the payment of additional services by healthcare workers, commissioning accredited private structures, and/or other actions identified by each LHA and aimed at recovering WLs. The lead LHA identified is the LHA City of Turin of the regional capital [15,16].

In Turin, a metropolitan city with nearly 900,000 residents, territorial healthcare is organized into four districts on a geographical basis: the North-West, the North-East, the South-West, and the South-East Districts [17]. In particular, the South-East District, the setting of this study as explained in the dedicated section, provides primary care services and the coordination of the various primary care activities with those of the departments and hospital facilities for 205,010 resident inhabitants [18].

In our facility, as in other settings, an automatic recall system using voice messages recorded through a centralized call centre is already in place to remind patients of scheduled appointments [5].

Despite this system, no-shows continue to challenge local healthcare resources. While no-show behaviour has been investigated internationally, there is limited evidence from Italian public healthcare systems at the district level, especially in relation to integrated demographic, economic, and service-level predictors. This study aims to thoroughly analyse the prevalence and factors contributing to no-shows. By understanding these determinants, we can develop and refine strategies to mitigate no-shows, ultimately enhancing resource use, improving care access, and reducing WLs.

## 2. Materials and Methods

### 2.1. Study Design and Data Collection

This retrospective study analysed all outpatient appointments within the South-East District of the “Città di Torino” Local Health Authority from 1 January 2022 to 31 December 2023. Data were extracted from the corporate IT system for outpatient appointments. Consequently, data did not contain sensitive or directly identifiable patient information. The extracted variables about the patients’ characteristics included gender, age (categorized into three groups: under 18, 18–65, and over 65), residency, and the presence of a co-payment exemption, specifying whether it was due to income conditions or clinical conditions. Residency was classified into mutually exclusive categories: “Turin” refers to the municipality of Turin; “Turin province” refers to the province of Turin excluding the city; “Piedmont” refers to the remaining areas of the Piedmont region, excluding the Turin province; “Italy” refers to other Italian regions outside Piedmont; and “Foreigners” refers to patients with residence abroad. Each patient was assigned to one category only.

Healthcare services were classified into the following categories: first access and others, including follow-up visits, diagnostic/instrumental procedures, and treatments. The priorities related to services were categorized according to the priority system used in the Italian NHS: urgent (to be performed within 72 h), short-term (within 10 days), deferrable (within 30 days for visits and 60 days for diagnostics), and schedulable (within 120 days) [14]. Extracted variables also included the co-payment value for each service, as established by the current tariff for outpatient healthcare services provided by NHS healthcare facilities. The healthcare service sites (Site 1, Site 2, and Site 3) refer to the three outpatient facilities of the South-East District. Both the request and service delivery dates were recorded to calculate waiting times.

This study was conducted in accordance with the STROBE (Strengthening the Reporting of Observational Studies in Epidemiology) guidelines, and the completed checklist is provided as Supplementary Material. This article is a revised and expanded version of a conference paper presented at the 17th European Public Health Conference, held in Lisbon on 12–15 November 2024 [19].

### 2.2. Statistical Analysis

Data were analysed using the Jamovi cloud platform (version 2.6). To provide a detailed overview of patient and appointment characteristics, descriptive statistics were applied to all relevant variables. Given the non-normal distribution of quantitative data (as determined by Shapiro–Wilk tests), results are presented as medians with interquartile ranges (IQRs). To evaluate differences between “show” and “no-show” groups, Pearson’s chi-square test was used for categorical variables, while the Mann–Whitney U-test was applied to continuous variables. These tests were chosen based on their robustness for comparing independent groups with non-parametric distributions, appropriate for the skewed data distributions often seen in healthcare datasets.

A binomial logistic regression model was used to identify predictors of no-shows. Predictor selection was grounded in the literature and logical independence of predictors rather than significance testing in order to reduce risks of parameter uncertainty, overfitting, and bias [20,21]. To manage missing data, 1695 (1.39%) observations with missing values in any variables were removed using listwise deletion. The logistic regression analysis was further reviewed for multicollinearity to ensure predictor independence. Model fit was assessed through deviance, Akaike information criterion (AIC), and McFadden’s pseudo-R^2^. Results are reported as odds ratios (ORs) with 95% confidence intervals (CIs) and corresponding *p*-values, with *p*-values < 0.05 deemed statistically significant.

## 3. Results

### 3.1. Bivariate Analysis

Table 1 presents a summary of healthcare appointments attended versus missed (“show” vs. “no-show”) within the South-East District of the “Città di Torino” Local Health Authority across 2022 and 2023. A total of 6198 appointments were recorded as no-shows out of 120,405 scheduled appointments, resulting in a no-show percentage of 5.1%. This percentage reflects the extent to which potentially deliverable healthcare services were affected by patient non-attendance.

The distribution of no-shows varied significantly across healthcare service sites. Site 1, which managed the highest volume of appointments, reported the lowest no-show percentage at 4.4%. In contrast, Site 2, with fewer overall appointments, exhibited the highest no-show percentage at 6.8%. Site 3 displayed an intermediate no-show percentage of 4.9%, placing it between the other two sites regarding patient attendance.

Regarding appointment type, “first access” services—typically comprising new patient visits—had a notably higher no-show percentage of 6.1% than the “others” category, which includes follow-up or diagnostic appointments (collectively at 4.7%). Moreover, the lowest no-show percentage was observed for urgent appointments (2.2%), while short-term appointments exhibited a slightly higher percentage (approximately 3.3%). In contrast, appointments categorized as deferrable or schedulable demonstrated higher no-show percentages of 6.3% and 5.1%, respectively.

### 3.2. Patient Characteristics and No-Show Percentages

**Figure 1:**
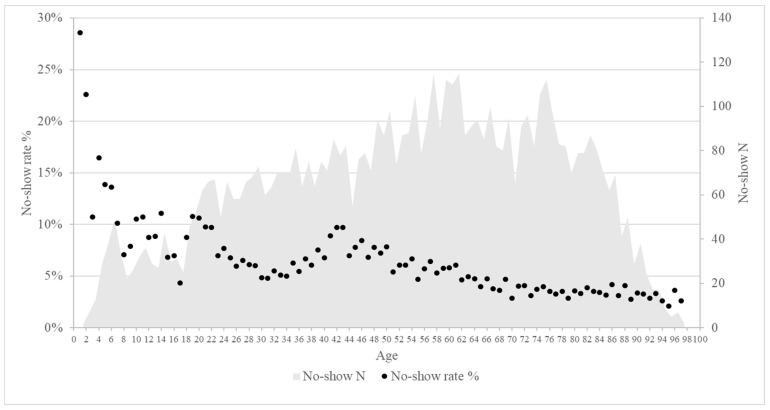
Scatter plot: on the *X*-axis, the age of the patients; on the *Y*-axis, the no-show percentage in percentage in the left and the no-show total number in the right.

Patient demographic characteristics also played a significant role in attendance patterns. Male patients had a slightly higher no-show percentage of 5.5% compared to females at 4.9%. Age also strongly influenced attendance: the median age of the “no-show” group was 54 (range: 35–71), while the median age of the “show” group was 64 (range: 44–77). Younger patients (<18 years) had the highest no-show percentage of 9.3%, while middle-aged adults (18–65 years) had a percentage of 6.4%, and the elderly (>65 years) had the lowest percentage at 3.6%. This demonstrated a trend whereby the percentage of no-shows decreased with increasing age (Figure 1).

In terms of residency, patients residing within the immediate Turin area or its province had no-show percentages of 5.0% and 5.1%, respectively. Patients from other parts of Piedmont or Italy as well as international residents showed progressively higher percentages, ranging from 6.7% to 11.4%. This trend suggests that geographic proximity and familiarity with local healthcare services may positively impact attendance.

Economic factors such as co-payment exemptions further influenced no-show percentages. Patients with clinical condition-based exemptions had the lowest no-show percentage at 4.0%. Those exempt due to income had a slightly higher no-show percentage (5.4%), while patients without any exemptions showed the highest percentage at 6.6%. However, the median co-payment value did not significantly impact attendance, while the median waiting period for no-show appointments was significantly longer compared to attended appointments.

### 3.3. Logistic Regression Analysis of Predictors for No-Show

Table 2 presents the results of the binomial logistic regression model, showing associations between selected variables and the likelihood of no-shows.

Patients accessing healthcare services at Site 2 were 1.52 times more likely to miss appointments than those at Site 1 (OR = 1.52, 95% CI: 1.42–1.62; *p* < 0.001). Similarly, patients accessing Site 3 showed higher odds of non-attendance compared to Site 1 (OR = 1.18, 95% CI: 1.10–1.27; *p* < 0.001). Regarding healthcare service categories, “first access” was associated with a higher odds of no-shows compared to the “others” category (OR = 1.11, 95% CI: 1.04–1.18; *p* < 0.001).

Priority levels revealed that short-term appointments had a lower odds of non-attendance than schedulable ones (OR = 0.86, 95% CI: 0.78–0.96; *p* = 0.005), while deferrable appointments showed the opposite pattern (OR = 1.22, 95% CI: 1.15–1.30; *p* < 0.001).

For patient characteristics, male patients had 1.17 times the odds of non-attendance compared to female patients (95% CI: 1.11–1.24; *p* < 0.001). Age comparisons indicated that patients aged 18–65 years and those under 18 were over twice as likely to miss appointments compared to those over 65 years (OR = 2.32, 95% CI: 2.17–2.47, *p* < 0.001; OR = 2.46, 95% CI: 2.20–2.74, *p* < 0.001, respectively).

Residency showed that foreign residents were 2.46 times more likely to miss appointments compared to Turin residents (OR = 2.46, 95% CI: 1.98–3.04; *p* < 0.001), while residents of Turin Province had lower odds (OR = 0.92, 95% CI: 0.86–0.99; *p* = 0.024).

Finally, each extra waiting day slightly increased the odds of missing an appointment (OR = 1.01, 95% CI: 1.01–1.01; *p* < 0.001).

The model showed a deviance of 46,167, an AIC of 46,203, and a McFadden’s R^2^ of 0.054.

## 4. Discussion

This study highlights important factors associated with no-show percentages across various healthcare service sites, with significant disparities suggesting that accessibility may play a crucial role in patient attendance. Lower no-show percentages observed at certain sites may stem from variables like higher reachability via public transportation, more effective management practices, or a more engaged patient population in terms of healthcare service utilization [22]. This underscores the value of optimizing site management and enhancing accessibility as potential strategies to reduce no-show percentages and improve healthcare service delivery overall. Regarding the influence of appointment urgency, our analysis indicates that short-term appointments (within 10 days) have significantly lower no-show percentages, reflecting both their critical nature and patients’ perceived need for these services. Conversely, deferrable and schedulable appointments exhibited higher no-show percentages, consistent with findings from other European studies [3] that have linked perceived clinical priority to attendance probability. The findings indicate that patients residing in Turin generally have lower no-show percentages, while percentages of non-attendance increase with distance from the healthcare facility, as other studies have already shown [3,8]. Interestingly, residents of the surrounding Turin Province exhibited a lower likelihood of missing appointments compared to city residents, as highlighted in the logistic regression model. The lower no-show rate in the provincial area compared to the city could be due to the use of the regional healthcare booking system (Centro Unificato Prenotazioni, CUP) by patients residing in the provincial area and could reflect greater scheduling discipline among patients needing to travel longer distances, suggesting a self-selection effect or higher perceived value of care. Conversely, the convenience of closer proximity for city residents might encourage procrastination, ultimately leading to higher percentages of no-shows compared to resident in the provincial area [23,24]. Moreover, foreign patients exhibited the highest likelihood of no-shows, highlighting potential barriers to healthcare access for this group. These challenges may include language barriers, limited familiarity with the healthcare system, and socioeconomic issues [3,25]. Addressing these barriers through targeted outreach and support programs could help lower no-show percentages and improve healthcare access for foreign residents.

Regarding the influence of age, elderly patients have significantly lower no-show percentages compared with younger age groups in our study. This trend might stem from higher healthcare needs among elderly patients, prompting them more likely to prioritize medical appointments [11,24]. Additionally, younger patients may be less familiar with the healthcare system, leading to higher no-show percentages among those attending their first appointment. A higher no-show percentage could reflect difficulties in navigating the system or identifying a true health need. This aligns with previous research showing a higher tendency for missed appointments among those new to the healthcare system [3]. Furthermore, this study highlighted that patients exempt from co-payments due to clinical conditions, a circumstance that may benefit patients with chronic diseases, have a lower percentage of no-shows at healthcare appointments. This lends further support to the hypothesis that individuals with greater awareness of their health condition tend to value healthcare services more highly.

The observed gender effect is also notable: in our study, male patients showed significantly higher odds of non-attendance (OR = 1.17; 95% CI 1.11–1.24). A similar pattern was reported in the large Danish register-based cohort analysed by Wolff et al., where male gender remained an independent risk factor after adjustment for age, diagnosis, and socioeconomic status (OR ≈ 1.35) [26]. Conversely, other European studies have found no significant gender differences, indicating that the impact of gender may be context-dependent [3]. This association may stem from known differences in health-seeking behaviours between men and women, with men being typically less engaged in preventive care and more reluctant to seek help [27]. From an economic perspective, our study indicates that patients exempt from co-payments on the basis of income as well as those without any exemption record higher no-show percentages than patients exempt for clinical conditions. A comparable pattern was observed in several studies [3], such as in the large Danish outpatient cohort analysed by Wolff et al., where receipt of social welfare benefits was independently associated with increased odds of non-attendance (OR ≈ 1.48) [26]. Since income-based exemption typically corresponds to lower socioeconomic status, it is possible that exemption status may influence attendance because some patients might be unaware of potential charges associated with missed appointments [4]. From a health-system standpoint, missed appointments in exempt groups translate into lost capacity and additional administrative costs. Reducing no-show percentages among exempt patients, perhaps through personalized reminders and improved communication, could help mitigate these financial losses and promote better resource utilization [5].

One limitation of this study is its observational design, which precludes the establishing of causal relationships between the analysed variables and no-show percentages. While associations can be identified, further experimental or longitudinal studies would be necessary to confirm causality. Another limitation is the potential for unmeasured confounding variables that could influence no-show percentages: factors such as patient education levels, employment status, transportation availability, distance from the healthcare site, and repeat no-show behaviour were not available in this study but could significantly impact appointment attendance [3]. Moreover, while income-based exemptions are determined according to defined income thresholds and can serve as an administrative indicator of low socioeconomic status, this measure does not fully capture the complexity of patients’ socioeconomic conditions. Additionally, the study was conducted within a specific regional healthcare system, which may limit the generalizability of the findings to other settings with different healthcare infrastructures and patient demographics. Finally, the logistic regression model’s metrics highlight limitations in fully capturing the complexity of factors contributing to no-shows. Expanding the dataset and the variables included in future studies could improve the model’s predictive accuracy and provide deeper insights.

## 5. Conclusions

In conclusion, the present study identifies several factors that contribute to the occurrence of no-shows in healthcare appointments. The accessibility and management of healthcare service sites, the urgency and nature of appointments, patient demographics, and socio-economic status all play a significant role in this regard. Patients aged 65 and over as well as those with short-term healthcare needs are less likely to miss appointments. In contrast, younger patients and those accessing healthcare for the first time are more likely to miss appointments. Socioeconomic factors such as income-based exemption also affect attendance, possibly due to a lack of awareness about the financial obligations of missed appointments. Furthermore, longer waiting times are associated with increased no-show percentages, emphasizing the need to reduce waiting times to enhance patient compliance. Building on these insights, several organizational-level interventions could be considered to improve attendance rates. First, integrating predictive analytics to identify high-risk no-show patients can allow staff to deploy targeted interventions such as personalized phone calls or SMS reminders. Second, implementing overbooking strategies cautiously and supported by robust predictive models could compensate for expected no-show rates without substantially increasing patient wait times or overloading staff. Finally, improving communication regarding the importance of attending appointments and clarifying the potential costs of non-attendance may enhance engagement, especially among patients exempt from co-payments who might perceive fewer consequences for missed visits

## Figures and Tables

**Table 1:** Bivariate analysis of healthcare activity divided by “no-show” and “show”.

Variable	No-Show *	Show *	*p*-Value	No-Show Percentage (%) **
**Characteristics of the healthcare services**				
**Healthcare services sites, n (%)**			<0.001 ^1^	
Site 1	2638 (42.6%)	57,929 (50.7%)		4.4%
Site 2	2262 (36.5%)	31,078 (27.2%)		6.8%
Site 3	1298 (20.9%)	25,200 (22.1%)		4.9%
**Healthcare services categories, n (%)**			<0.001 ^1^	
First access	2441 (39.4%)	37,430 (32.8%)		6.1%
Others	3757 (60.6%)	76,777 (67.2%)		4.7%
**Healthcare services priority, n (%)**			<0.001 ^1^	
Urgent	7 (0.1%)	315 (0.3%)		2.2%
Short-term	474 (7.6%)	14,065 (12.3%)		3.3%
Deferrable	1702 (27.5%)	25,486 (22.3%)		6.3%
Schedulable	4015 (64.8%)	74,341 (65.1%)		5.1%
**Specialty type, n (%)**			<0.001 ^1^	
Anaesthesiology/Analgesia	74 (1.2%)	2174 (1.9%)		3.3%
Cardiology	1466 (23.7%)	29,472 (25.8%)		4.7%
Dentistry and Stomatology	821 (13.3%)	7231 (6.3%)		10.2%
Dermatology	257 (4.2%)	3756 (3.3%)		6.4%
Diabetology	307 (5%)	3623 (3.2%)		7.8%
Endocrinology	309 (5%)	4601 (4%)		6.3%
General Surgery	94 (1.5%)	1744 (1.5%)		5.1%
Geriatrics	68 (1.1%)	1874 (1.6%)		3.5%
Neurology	278 (4.5%)	4500 (3.9%)		5.8%
Obstetrics and Gynaecology	502 (8.1%)	11,969 (10.5%)		4%
Ophthalmology	600 (9.7%)	10,305 (9%)		5.5%
Orthopaedics and Traumatology	553 (8.9%)	7071 (6.2%)		7.3%
Otorhinolaryngology	556 (9%)	7434 (6.5%)		7%
Rheumatology	86 (1.4%)	1622 (1.4%)		5%
Urology	118 (1.9%)	4148 (3.6%)		2.8%
Other Specialties	109 (1.8%)	12,683 (11.1%)		0.9%
**Co-payment value in euros, median (IQR)**	20.7 (12.9–20.7)	20.7 (12.9–20.7)	NS ^2^	/
**Waiting days, median (IQR)**	70 (21–130)	21 (6–75)	<0.001 ^2^	/
**Patients’ characteristics**				
**Gender, n (%)**			<0.001 ^1^	
Male	2705 (43.7%)	46,790 (41%)		5.5%
Female	3493 (56.3%)	67,417 (59%)		4.9%
**Age, n (%)**			<0.001 ^1^	
<18 years	472 (7.6%)	4592 (4.0%)		9.3%
18–65 years	3634 (58.6%)	53,496 (46.8%)		6.4%
>65 years	2092 (33.8%)	56,119 (49.1%)		3.6%
**Residency, n (%)**			<0.001 ^1^	
Turin	4897 (79%)	92,343 (80.9%)		5%
Turin Province	949 (15.3%)	17,784 (15.6%)		5.1%
Piedmont	100 (1.6%)	1402 (1.2%)		6.7%
Italy	150 (2.4%)	1887 (1.7%)		7.4%
Abroad	102 (1.6%)	791 (0.7%)		11.4%
**Co-payment exemption, n (%)**			<0.001 ^1^	
No	2123 (34.3%)	29,908 (26.2%)		6.6%
Yes, by income	2041 (32.9%)	35,689 (31.3%)		5.4%
Yes, due to clinical conditions	2034 (32.8%)	48,610 (42.6%)		4%
**Total**	**N = 6198**	**N = 114,207**		**5.1%**

^1^ Pearson’s chi-square test; ^2^ Mann–Whitney U-test. IQR = Interquartile range; NS = not significant. * Column percentages related to each variable are shown in these columns. ** Row percentages related to each variable are shown in this column.

**Table 2:** Predictors of No-Show: results from binomial logistic regression.

Variable	OR *	95% CI *	*p*-Value *
**Characteristics of the healthcare services**			
**Healthcare services sites**			
2–1	1.52	1.42–1.62	<0.001
3–1	1.18	1.10–1.27	<0.001
**Healthcare services categories**			
First access–Others	1.11	1.04–1.18	<0.001
**Healthcare services priority**			
Urgent–Schedulable	0.61	0.29–1.30	NS
Short-term–Schedulable	0.86	0.78–0.96	0.005
Deferrable–Schedulable	1.22	1.15–1.30	<0.001
**Co-payment value in euros**	1.00	1.00–1.00	0.014
**Waiting days**	1.01	1.01–1.01	<0.001
**Patients’ characteristics**			
**Gender**			
Male–Female	1.17	1.11–1.24	<0.001
**Age**			
18–65 years–>65 years	2.32	2.17–2.47	<0.001
<18 years–>65 years	2.46	2.20–2.74	<0.001
**Residency**			
Foreigner–Turin	2.46	1.98–3.04	<0.001
Italian–Turin	1.31	1.10–1.55	0.002
Piedmont–Turin	1.15	0.94–1.42	NS
Turin Province–Turin	0.92	0.86–0.99	0.024
**Co-payment exemption**			
No–Yes, due to clinical conditions	1.36	1.27–1.46	<0.001
Yes, by income–Yes, due to clinical conditions	1.82	1.69–1.95	<0.001

* OR = odds ratio; CI = confidence interval; NS = not significant.

## Data Availability

The data that support the findings of this study are available from the corresponding author upon reasonable request.

## Supplementary Materials

The following supporting information can be downloaded at https://www.mdpi.com/article/10.3390/healthcare13151869/s1. Supplementary Material S1. STROBE Statement.
